# Supplementary material for: An In Vivo Platform for Rebuilding Functional Neocortical Tissue
Source: Bioengineering (Basel). 2023 Feb 16;10(2):263. doi: 10.3390/bioengineering10020263 (PMC9952056; doi:10.3390/bioengineering10020263)
Supplement: Supplementary file 1 [file bioengineering-10-00263-s001.zip › bioengineering-2161369-SM.pdf]

bioengineering

MDPI

Article

An in vivo platform for rebuilding functional neocortical tissue

Alexandra Quezada[1,2,¥], Claire Ward[1,¥], Edward R. Bader[3], Pavlo Zolotavin[4], Esra Altun[4], Sarah Hong[1], Nathan Killian[3], Chong Xie[4], Renata Batista-Brito[1§], Jean M. Hébert[1,2,5,§,]

1 Department of Neuroscience, Albert Einstein College of Medicine, Bronx, NY, USA
2 Stem Cell Institute, Albert Einstein College of Medicine, Bronx, NY, USA
3 Department of Neurological Surgery, Albert Einstein College of Medicine, Bronx, NY, USA
4 Department of Electrical and Computer Engineering, Rice University, Houston, TX, USA
5 Department of Genetics, Albert Einstein College of Medicine, Bronx, NY, USA
* Correspondence: jean.hebert@einsteinmed.edu
¥ Co-first authors
§ Co-senior authors

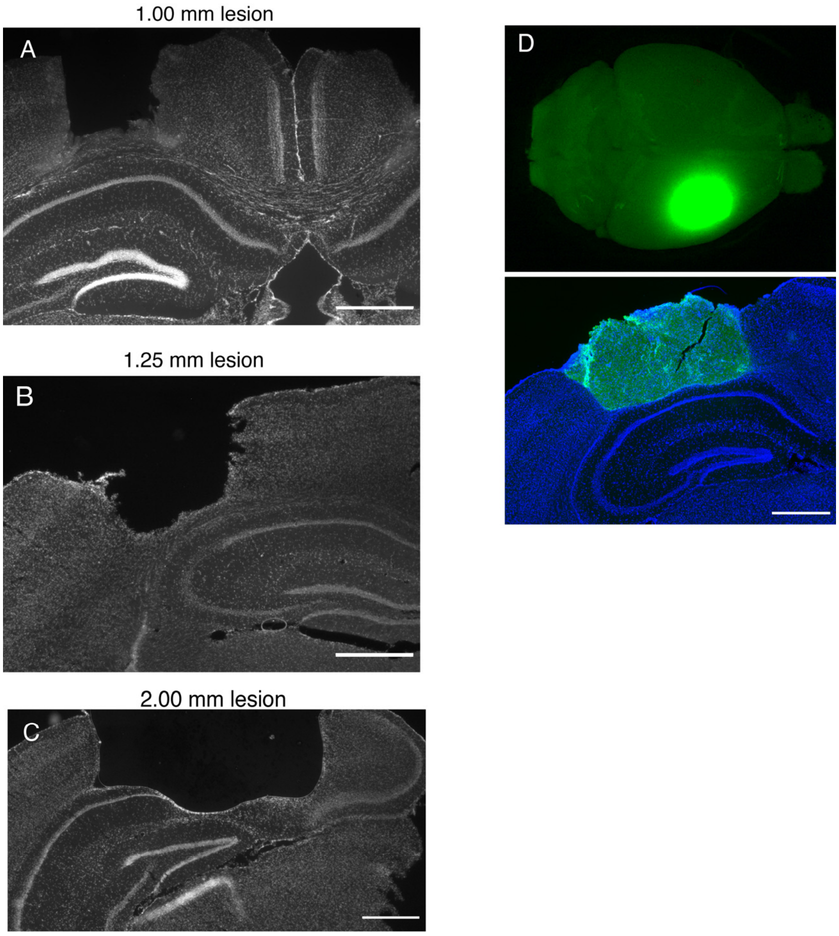

Figure S1. Cortical lesions can differ in size without obvious impacts on engraftment. A. 1 mm cortical lesion (Scale bar=1 mm). B. 1.25 mm cortical lesion (Scale bar=1 mm). C. 2 mm cortical lesion (Scale bar=1 mm). D. Representative image of a 2 mm graft. Top panel, GFP-labeled graft in whole mouse brain. Bottom panel, immunofluorescence image of coronal section of graft (Scale bar, 1 mm).

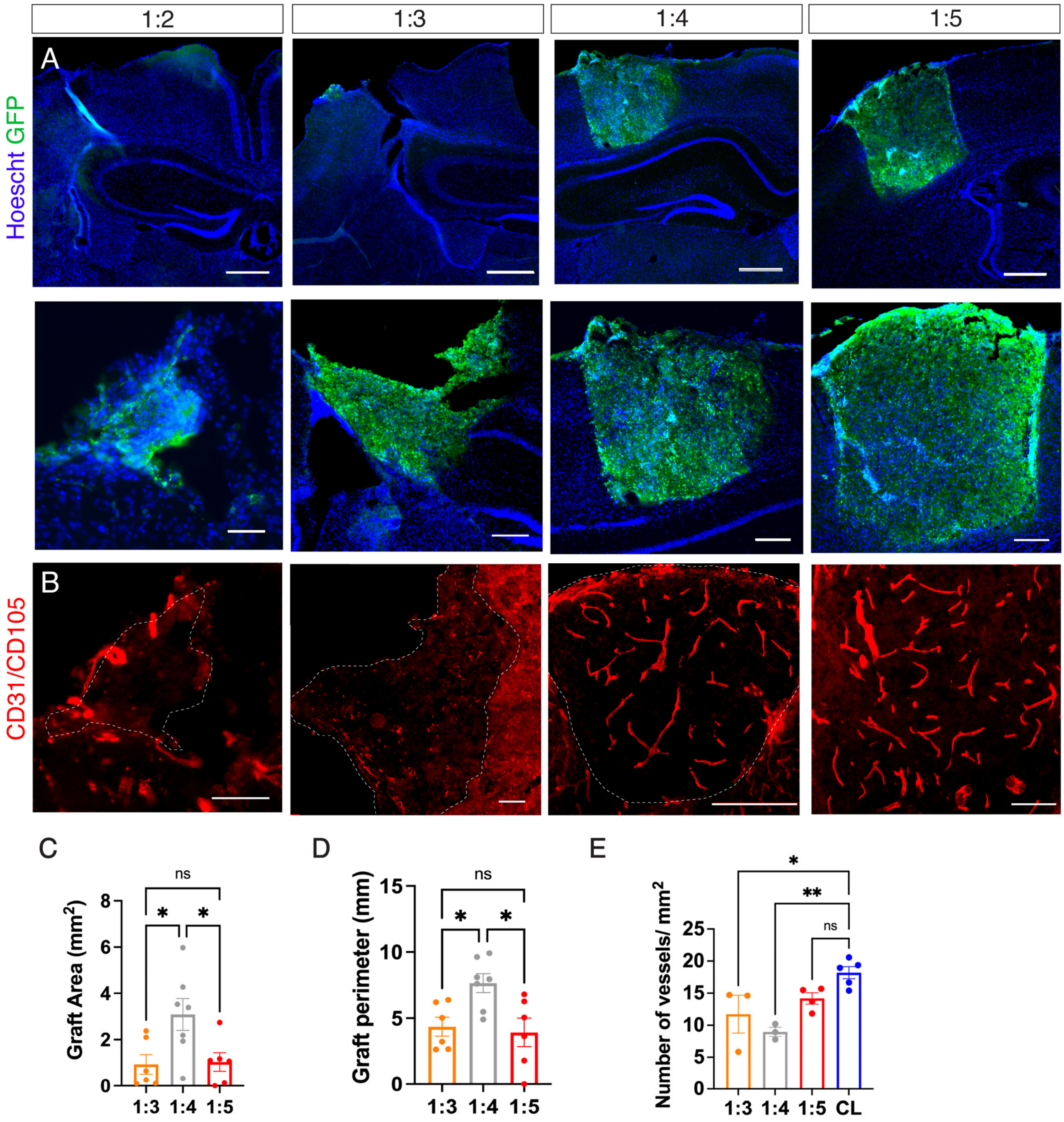

Figure S2. The quality of grafts depends on scaffold concentration. A. Representative immunofluorescence images of grafts with volume ratios of Matrigel:media from left to right of 1:2, 1:3, 1:4, 1:5. Top row, low magnification (Scale bars from left to right = 800 µm, 500 µm, 1 mm, 1 mm). Bottom row, higher magnification (Scale bars from left to right = 100 µm, 150 µm, 300 µm, 300 µm). B. Representative immunofluorescence images of graft vasculature for each Matrigel:media ratio (Scale bars from left to right = 100 µm, 150 µm, 500 µm, 200 µm). C. Quantification of graft area. Grafts with 1:4 dilution (N = 7, mean = 3.01 mm^2), were significantly larger than 1:3, (N = 6, mean = 0.93 mm^2, p = 0.03) and 1:5, (N = 6, mean = 1.03 mm^2, p = 0.04). D. Quantification of graft perimeter. Grafts with the 1:4 dilution (N = 7, mean = 7.66 mm), shared a significantly longer border with the host tissue than the 1:3 (N = 6, mean = 4.36 mm, p = 0.03) and 1:5mm^2 (N = 6, mean=3.92 mm, p = 00.02). E. Quantification of vessel density in grafts. Contralateral cortex had significantly higher vessel density than grafts with the 1:3 dilution (N=3, mean= 11.72 mm^2, p=0.02) and 1:4 dilution (N = 3, mean = 8.97 mm^2, p = 0.002). Grafts with the 1:5 dilution (N = 4, mean = 14.18 mm^2, p = 0.13) were not statistically different than contralateral cortex.
